# Molecular analysis of the vaginal response to estrogens in the ovariectomized rat and postmenopausal woman

**DOI:** 10.1186/1755-8794-1-27

**Published:** 2008-06-25

**Authors:** Scott A Jelinsky, Sung E Choe, Judy S Crabtree, Monette M Cotreau, Ewa Wilson, Kathryn Saraf, Andrew J Dorner, Eugene L Brown, Bryan J Peano, Xiaochun Zhang, Richard C Winneker, Heather A Harris

**Affiliations:** 1 Biological Technologies, Wyeth Research, Cambridge, USA; 2 Women's Health and Musculoskeletal Biology, Wyeth Research, Collegeville, USA; 3 Translational Research, Wyeth Research, Cambridge, USA

## Abstract

**Background:**

Vaginal atrophy (VA) is the thinning of the vaginal epithelial lining, typically the result of lowered estrogen levels during menopause. Some of the consequences of VA include increased susceptibility to bacterial infection, pain during sexual intercourse, and vaginal burning or itching. Although estrogen treatment is highly effective, alternative therapies are also desired for women who are not candidates for post-menopausal hormone therapy (HT). The ovariectomized (OVX) rat is widely accepted as an appropriate animal model for many estrogen-dependent responses in humans; however, since reproductive biology can vary significantly between mammalian systems, this study examined how well the OVX rat recapitulates human biology.

**Methods:**

We analyzed 19 vaginal biopsies from human subjects pre and post 3-month 17β-estradiol treated by expression profiling. Data were compared to transcriptional profiling generated from vaginal samples obtained from ovariectomized rats treated with 17β-estradiol for 6 hrs, 3 days or 5 days. The level of differential expression between pre- vs. post- estrogen treatment was calculated for each of the human and OVX rat datasets. Probe sets corresponding to orthologous rat and human genes were mapped to each other using NCBI Homologene.

**Results:**

A positive correlation was observed between the rat and human responses to estrogen. Genes belonging to several biological pathways and GO categories were similarly differentially expressed in rat and human. A large number of the coordinately regulated biological processes are already known to be involved in human VA, such as inflammation, epithelial development, and EGF pathway activation.

**Conclusion:**

At the transcriptional level, there is evidence of significant overlap of the effects of estrogen treatment between the OVX rat and human VA samples.

## Background

In many women, declining estrogen levels at menopause lead to a number of physiological changes, including vasomotor instability (hot flushes), mood swings, decreased libido, vaginal atrophy (VA), serum lipid changes and the increased risk of developing osteoporosis. While progestin and/or estrogen therapy are clinically proven to ameliorate some of these symptoms, these medications are contraindicated in some populations, and other women choose not to take them. Because of these issues, recent work has focused on developing more specific, and in many cases, nonhormonal alternatives to traditional post-menopausal hormone therapy. These alternatives have focused largely on reduction of hot flushes [1,2] and the treatment/prevention of osteoporosis [3-5].

VA affects up to 40% of postmenopausal women, with those with an active sex life being more apt to complain of symptoms (see [6] for review). Its hallmarks are thinning epithelia and increased vaginal pH. Symptoms include dryness, burning, itching, decreased lubrication during sexual stimulation and painful intercourse. In extreme cases the epithelium is absent and adhesions form in the vaginal vault, preventing clinical exams and drainage of fluid [7]. Currently, estrogens (both systemically and locally administered) are the only approved medical therapies for treating VA. Lubricants can relieve some of the symptoms of VA, but do not treat the underlying cause.

Despite the fact that the vagina is a classic estrogen-responsive organ, it has received little attention compared to the adjacent uterus. Uterine responses to estrogens on the reproductive cycle have been well-characterized and several expression profiles have been generated [8-10], but no analogous studies have been performed on the vagina, aside from a recent report that examines the vaginal effects of neonatal diethylstilbestrol exposure [11].

Our ultimate goal is to develop nonhormonal therapies for VA in order to provide additional treatment options for post-menopausal women. Key to evaluating new compounds is the development of preclinical animal models, preferably in the rat or mouse. Drawing on the extensive studies of the rat uterine response to estrogens for the development of selective estrogen receptor modulators (SERMs) [12-15], we constructed an analogous model of rat vaginal responses. This report details the time course of mRNA changes elicited by 17β-estradiol in the ovariectomized (OVX) rat vaginal vault and compares these responses to those seen in the human vaginal epithelium after three months of 17β-estradiol patch therapy.

## Methods

### Animals care/tissue collection/dosing

All animal studies were approved by the Institutional Animal Care and Use Committee of Wyeth Research, Collegeville, PA and were conducted in accordance with Association for the Assessment and Accreditation of Laboratory Animal Care (AAALAC) guidelines. Sprague Dawley rats (Taconic, Germantown NY) were OVX at 8 weeks of age and rested for about 1 week before administration of daily subcutaneous doses of 17β-estradiol (20 μg/kg) or vehicle (50% DMSO/50% 1× Dulbecco's PBS). Animals were euthanized after 6 hrs, 3 days and 5 days of treatment. For the 3- and 5-day timepoints, animals were euthanized 6 hours after the last dose. At necropsy, the vaginal vault was excised and trimmed of adherent tissue (e.g. the urethra). Typically, organs were trimmed just below the cervix and above the external orifice of the vagina before flash freezing in liquid nitrogen.

For the studies using betacellulin, 8-week-old female Sprague Dawley rats were OVX and allowed to recover from surgery for 10 days prior to treatment. Rats were dosed intravaginally for 5 days with mouse betacellulin (200 nM; R&D Systems, Minneapolis MN) once daily using a carbomer-934P-based vehicle (pH 7.0) and a 0.5 inch long, 18 gauge ball-tipped gavage needle. Following euthanasia by CO_2 _asphyxiation and pneumothorax, the vaginal vault was removed, flattened on an index card and fixed in 10% buffered formalin (VWR International, West Chester, PA) for 16–72 hours. Vaginas were equilibrated in 15% sucrose for 3 hours prior to embedding in OCT (Sigma, St Louis MO) and cryosectioning. Ten micron sections were stained with hematoxylin and eosin (both from Sigma) for histological analysis.

### Human tissue collection

A clinical study was initiated to collect 3 mm vaginal biopsy specimens from post-menopausal women clinically diagnosed with VA. The diagnosis of VA was confirmed by a vaginal maturation index (VMI) containing ≤5% superficial cells and vaginal pH > 5.0. Patients were treated for 3 months with 17β-estradiol, delivered via a skin patch, at a dose of 0.05 mg/day. All 19 subjects had increased VMI (>5%) and/or reduced pH (< or = 5) following treatment. Vaginal biopsies were collected on day 1 prior to the initiation of treatment with 17β-estradiol and on the last day of treatment (day 85). Samples were immediately frozen on dry ice and stored at -80°C until RNA isolation. This study was conducted at the Ohio State University, Division of Clinical Pharmacology (Columbus, Ohio). Written informed consent was obtained from all subjects prior to any study-related procedure. Western Institutional Review Board (Olympia, WA) approved the protocol and informed consent. The details of the clinical analyses have been reported elsewhere [16].

### RNA isolation

Each rat vagina was homogenized in 3 mL of TRIzol reagent (Invitrogen, Carlsbad CA) using a Polytron PT3000 grinder (Brinkmann, Westbury NY) at a setting of 12–15 for 1 min. Subsequent RNA extraction was as described previously [17]. For human samples, RNA extraction was performed according to a modified RNeasy mini kit method. (Qiagen, Valencia, CA) [16].

### qRT-PCR analysis

ComplementaryDNA was amplified from 50 ng total RNA with the Ovation RNA Amplification System (Nugen, San Carlos CA). Real-time PCR reactions (qRT-PCR) were conducted using the TaqMan^® ^Universal PCR Master Mix (Applied Biosystems). Forward and reverse primers (10 μM) and 20 μM labeled probe was mixed with 2× PCR master mix in a total volume of 15 ul and added to 50 ng cDNA in final reaction volume of 25 μl.

PCR reactions were run on an automated fluorometer (ABI Prism 7000 Sequence Detection System, Applied Biosystems) in a 96-well format. PCR conditions for all reactions were as follows: 1 cycle of 50°C for 2 min., 95°C for 10 min. followed by 40 cycles of 95°C for 15 sec. and 60°C for 1 min. Relative expression was determined by using the C_T _method (Pontius et al., 2003) using Sequence Detector software, version 1.6.3. Complementary DNAs of each sample were evaluated in triplicate. Results were normalized to a control gene, SFSR10 RNA, which has been shown to have very consistent expression across all patient samples by microarray analysis [16].

The primer sequences for each transcript are as follows:

BTC: forward primer, 5'-GGTGTGAGAGAGTTGACTTGTTTTACC-3'; reverse primer, 5'-TGCTATCAAACAAATCACCAGAATC-3'; probe, 5'-TGTCCTCTGTCTCCTCT-3'

SFSR10: forward primer, 5'-AAGGGAACACTATACCTGTCATGGA-3'; reverse primer, 5'-GGTAAGCAAAGGACCTGAAAATAATATT-3'; probe, 5'-AAGACTTTGCCTGTTCAT-'

### Microarray processing

For rat vaginal samples, 5 μg of total RNA were used to generate biotin labeled cRNA using an oligo T7 primer in a reverse transcription reaction followed by *in vitro *transcription reaction with biotin labeled UTP and CTP. Ten μg of cRNA were fragmented and hybridized to RAE230A and RAE230B arrays (Affymetrix, Santa Clara, CA). For human biopsies, RNA was labeled using Ovation™ Biotin System (Nugen, San Francisco, CA). Labeled cDNA (2.5 μg) was fragmented and hybridized to HGU133 Plus 2.0 arrays (Affymetrix, Santa Clara, CA) [16]. Hybridized arrays were stained according to manufacturer's protocols on a Fluidics Station 450 and scanned on an Affymetrix scanner 3000. All array images were visually inspected for defects and quality. Arrays with excessive background, low signal intensity, or major defects within the array were eliminated from further analysis. Signal values were determined using Gene Chip Operating System 1.0 (GCOS, Affymetrix). For each array, all probe sets were normalized to a mean signal intensity value of 100. The default GCOS statistical values were used for all analysis. Signal values and absolute detection calls were imported into Genesis 2.0 (GeneLogic, Gaithersburg, MD) for analysis. The complete data set is available at the Gene Expression Omnibus as GSE11622 [18].

### Determination of probe sets corresponding to homologous gene pairs

Mapping of RAE230 to HGU133 plus 2.0 probe sets was based on NCBI's Homologene (clusters of orthologous genes). First, all qualifiers were annotated with unigene accession numbers through BioExpress (GeneLogic). These accession numbers then were used to map orthologous sequences through NCBI Homologene [19]. Commonly one probe set from one species maps to multiple probe sets from a second species. To resolve multiple mappings, clusters of orthologous probe sets were generated as follows. For each human probe set, all corresponding rat probe sets were identified and grouped into a "cluster". Human probe sets that map to the same set of rat probes were clustered. For each human cluster, the corresponding locuslink ID(s) were identified. Clusters with multiple Locuslink ID annotations were discarded. Approximately 10% of the human probe sets clustered to multiple Locuslink IDs. Clustering identified 9093 groups, spanning 22460 human probe set IDs and 13219 rat probe set IDs. Each cluster was annotated (assigned GO/KEGG etc categories) based on the human Locuslink ID in that cluster.

### Identification of regulated genes

Identification of estrogen-regulated genes was done in two independent ways. In the first method, probe sets were considered estrogen regulated if the following conditions were met: 1) A gene was expressed greater than 50 signal units, 2) the percentage of samples with a Present (P) call as determined by GCOS default settings was greater than or equal to 66% in any sample condition, 3) the fold change between all estrogen treated and vehicle treated samples was at least 1.7, and 4) the p-value based on an Wilcoxon t-test was ≤0.01 (rat) or based on a Wilcoxon paired test was <0.01 (human). Over 5300 rat probe sets and 1668 human probe sets met these conditions. The second method used the 9093 clusters identified above. Arrays were subjected to a Loess transformation and the CyberT-statistics were calculated. False discovery rate estimates (q-values) were estimated using a null distribution of CyberT's from permuted data.

### Identification of significantly regulated gene sets

Statistical identification of significantly regulated biological pathways was implemented using a modified approach of Tian et al. [20]. 7798 gene sets were tested in the sigpathway analysis. These were derived from the follow sources: 1) GO annotations (from Affymetrix) [21], 2) KEGG/GenMAPP annotations (from Affymetrix) [21], 3) Functional categories (from MSigDB), 4) Genes that are located in close proximity along the chromosome (from MSigDB), 5) Genes containing instances of a regulatory motif of interest (from MSigDB), and 6) Genes that are co-regulated with a given gene in a variety of datasets (from MSigDB). Gene sets from MSigDB were derived from pathway databases (Biocarta, KEGG, and BioCyc) and pathway-specific microarray annotations [22], as well as >5,000 gene sets from Gene Ontology. Only gene sets in which the number of genes was greater or equal to 10 and less than or equal to 400 were used for further analysis. Each gene set was assigned a score of differential expression between two conditions (essentially, the sum of t-statistics for each gene within the set), and the false discovery rate (Q) associated with this score was calculated via two separate permutation tests (permuting gene labels (Q1) and sample labels (Q2)). A gene set was considered significant if its two false discovery rates are below 0.15 (Q1 <= 0.15 and Q2 <= 0.15) for all 4 comparisons. 45 gene sets passed these criteria.

## Results

### Estrogen-responsive genes in the rat vaginal vault

Eight-week-old rats were OVX and rested for about 1 week to mimic the loss of estrogen commonly seen in postmenopausal women. Animals were then treated with 17β-estradiol for 6 hrs, 3 days or 5 days following surgery. Rat vaginal vault tissue was collected, mRNA prepared and estrogen regulated genes identified through transcriptional profiling. Three time points were chosen for analysis to capture the range of responses to estradiol: six hours, representing the early phase of the estrogen-signaling cascade, five days, at which time vaginal histology is completely restored, and three days, an intermediate time point. We found the vaginal tissue to be extremely responsive to 17β-estradiol, with over 5300 unique probe sets (~17% of the probe sets monitored) showing differential expression in at least one time point (fold change > 1.7, p-value <0.01, Table 1). Surprisingly, a majority (> 63%) of these genes were down regulated after estradiol treatment. We find regulation of many known estrogen target genes [23-25] including calbindin-3 (77 fold), kallikrein 6 (33 fold), arginase 1 (27 fold), adenosine deaminase (14.6 fold), progesterone receptor (3.8 fold), cathepsin D (2.3 fold) and trefoil factor (2.1 fold) that are up regulated in rat vagina tissue.

**Table 1:** Number of probe sets regulated by 17-β estradiol in rat vaginal tissue

	Up regulated	Down Regulated	Total
Genes regulated at 5 days	1305	2395	3700
Genes regulated at 3 days	1513	2364	3877
Genes regulated at 6 hours	631	1155	1786

### Differentially expressed genes in the human

Human vaginal biopsy specimens from 19 postmenopausal women clinically diagnosed with VA were collected. Participants in the study were treated for 3 months with 17β-estradiol, delivered via a skin patch, with vaginal biopsies collected prior to the initiation of treatment and on the last day of treatment. Microarray analysis of these biopsies resulted in 1668 genes regulated by at least 1.7 fold (772 up regulated and 896 down regulated, Wilcoxon paired t-test p < 0.01). The details of this analysis are described elsewhere [16]. Like the rat vaginal samples, we find the human vagina to be very responsive to estradiol although we see fewer genes regulated in the human samples. The difference in the number of significant genes could be the result of several factors, such as the greater variability (in genetics, age, etc) amongst the human patients in comparison to the inbred rats, or the fact that the human biopsies contained primarily epithelial cells, while the rat samples were the entire vaginal vaults.

### Mapping of RAE230 to HGU133 Plus 2.0 probe sets

**Figure 1:**
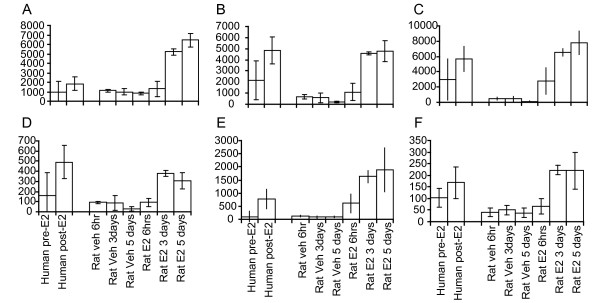
**Expression of genes with similar expression patterns in both human and rat.** Messenger RNA levels for (A) *keratin 5b*, (B) *Spink5*, (C) *Sprr1a*, (D) *FLJ21511*, (E) *Desmoglein 5 *and (F) *Abhd5 *are shown for each individual treatment.

Our goal was to determine how closely the OVX rat model of VA mimics the human disorder. Our first step required identifying pairs of orthologous genes, and finding the rat (RAE230) and human (HG-U133 plus 2.0) probe sets corresponding to them. In order to do this, probe set annotations and ortholog assignments were obtained from BioExpress 2.0 (GeneLogic) and NCBI Homologene, respectively. When multiple probe sets on a chip query the same gene, a per-gene score of differential expression was calculated by taking the average t-statistic for those probe sets (see Methods for details). In total, we identified 9093 pairs of orthologous genes that were represented on both chipsets, and for each pair, calculated a score of differential expression for each rat and human. These 9093 pairs spanned 22460 human probe set IDs and 13219 rat probe set IDs. Each orthologous gene pair was annotated (assigned GO/KEGG categories) based on the human LocusLink ID for that pair. Of the 9093 pairs, 1102 (554 up/548 down, q < 0.05) were regulated in the human data set; of these, 707 (349 up/358 down, q < 0.05) were regulated in the rat after 5 days treatment and 558 (79%, 285 up/273 down) were coordinately regulated in both species. The top co-regulated genes include desmoglein, growth arrest and DNA-damage-inducible alpha, 3-hydroxy-3-methylglutaryl-Coenzyme A synthase 1 and ATPase, H+ transporting, lysosomal 34 kDa, V1 subunit D and expression of these genes is depicted in Figure 1. Table 2 and Table 3 contain the list of top co-regulated genes for induced and repressed genes.

**Table 2:** Top coregulated clusters of genes

		Human cluster		Rat cluster	
		
Gene Symbol	Gene Description	t*	q**	t*	q**
DSG1	desmoglein 1	6.5	0	12.8	0
GADD45A	growth arrest and DNA-damage-inducible, alpha	6.8	0	5.1	0
HMGCS1	3-hydroxy-3-methylglutaryl-Coenzyme A synthase 1	5.2	0.0002	9.5	0
SC4MOL	sterol-C4-methyl oxidase-like	4.1	0.0025	9.2	0
FAM46B	family with sequence similarity 46, member B	2.4	0.0896	10.2	0
CRABP2	cellular retinoic acid binding 2	4.0	0.0029	12.6	0
CBR3	carbonyl reductase 3	5.2	0.0002	6.5	0
SLURP1	secreted LY6/PLAUR domain	4.4	0.0014	9.1	0
ATP6V1D	ATPase, H+ transporting, lysosomal, V1 subunit D	4.4	0.0011	9.5	0
KLF4	Kruppel-like factor 4	4.3	0.0016	7.5	0
GPT2	glutamic pyruvate transaminase	3.3	0.0175	8.7	0
CLIC3	chloride intracellular channel 3	2.8	0.0481	9.2	0
MAL2	T-cell differentiation protein 2	4.2	0.002	7.9	0
S100A10	S100 calcium binding protein A10	3.7	0.0067	9.1	0
FLJ12151	hypothetical protein FLJ12151	4.7	0.0006	6.4	0
HMGCR	3-hydroxy-3-methylglutaryl-Coenzyme A reductase	4.3	0.0016	5.7	0
MAFB	musculoaponeurotic fibrosarcoma homolog B	3.7	0.0071	5.7	0
RAB10	RAB10, member RAS oncogene family	3.5	0.0119	8.7	0
SERPINB2	serine proteinase inhibitor, clade B 2	3.4	0.0137	7.9	0
SQLE	Squalene epoxidase	2.8	0.0471	8.4	0
AVPI1	arginine vasopressin-induced 1	4.4	0.0012	6.8	0
AIM1L	absent in melanoma 1-like	2.6	0.0625	8.8	0
BNIP3	BCL2/adenovirus E1B 19 kDa interacting protein 3	6.3	0	5.2	0
BZW1	basic leucine zipper and W2 domains 1	3.9	0.0038	6.3	0
LOC58489	hypothetical protein	2.9	0.0386	8.6	0
AACS	acetoacetyl-CoA synthetase	4.1	0.0024	4.7	0
MGC13102	hypothetical protein MGC13102	3.7	0.0068	7.6	0
ETF1	eukaryotic translation termination factor 1	2.8	0.0438	8.0	0
C4.4A	GPI-anchored metastasis-associated protein homolog	3.0	0.0327	9.2	0
HMOX1	heme oxygenase 1	5.5	0.0002	5.1	0
KCTD11	K+ channel tetramerisation domain containing 11	4.7	0.0006	3.8	0
CDK7	cyclin-dependent kinase 7	5.9	0.0002	2.4	0.02
JUNB	jun B proto-oncogene	3.5	0.0113	5.7	0
RIOK3	RIO kinase 3	4.7	0.0006	4.6	0
ME1	malic enzyme 1	4.6	0.0007	4.6	0
YWHAZ	tyrosine 3-monooxygenase activation protein, zeta	2.5	0.0779	9.0	0

t* Average t-statistic

q** Average q-value

**Table 3:** Top coregulated clusters of genes

		Human cluster		Rat cluster	
		
Gene Symbol	Gene Description	t*	q**	t*	q**
TXNIP	thioredoxin interacting protein	-5.5	0.0002	-12.5	0
RBP1	retinol binding protein 1, cellular	-4.2	0.0021	-15.1	0
IGFBP3	insulin-like growth factor binding protein 3	-4.6	0.0007	-10.0	0
IFITM3	interferon induced transmembrane protein 3 (1–8 U)	-5.2	0.0002	-9.0	0
DHRS8	dehydrogenase/reductase (SDR family) member 8	-5.4	0.0002	-5.6	0
LDHB	lactate dehydrogenase B	-4.6	0.0006	-6.1	0
LAPTM4B	lysosomal associated protein transmembrane 4 beta	-4.5	0.0008	-8.3	0
MUC1	mucin 1, transmembrane	-5.7	0.0002	-5.2	0
SFRP2	secreted frizzled-related protein 2	-5.4	0.0002	-5.8	0
IFITM1	interferon induced transmembrane protein 1 (9–27)	-4.2	0.0019	-6.8	0
CD74	CD74 antigen	-4.3	0.0015	-8.2	0
LR8	LR8 protein	-3.6	0.0076	-7.6	0
B2M	beta-2-microglobulin	-4.1	0.0025	-8.0	0
PPP1R1B	protein phosphatase 1, regulatory (inhibitor) subunit 1B	-4.8	0.0006	-6.1	0
PRKACB	protein kinase, cAMP-dependent, catalytic, beta	-5.2	0.0003	-4.2	0
TSPAN3	tetraspanin 3	-4.4	0.0014	-6.5	0
PROM1	prominin 1	-2.8	0.0452	-9.0	0
TMC4	transmembrane channel-like 4	-3.8	0.0045	-8.0	0
ITM2C	integral membrane protein 2C	-3.0	0.0343	-8.0	0
DHRS3	dehydrogenase/reductase (SDR family) member 3	-3.6	0.0079	-5.9	0
ITM2B	integral membrane protein 2B	-2.5	0.0778	-9.6	0
VIM	vimentin	-2.7	0.0503	-9.6	0
NPC2	Niemann-Pick disease, type C2	-4.0	0.0029	-6.5	0
AXL	AXL receptor tyrosine kinase	-4.4	0.0012	-4.9	0
HLA-DRA	major histocompatibility complex, class II, DR alpha	-3.3	0.0153	-8.5	0
EEF1A1	Eukaryotic translation elongation factor 1 alpha 1	-4.1	0.0023	-5.1	0
FOXQ1	forkhead box Q1	-3.5	0.0116	-6.1	0
IFNGR1	interferon gamma receptor 1	-2.5	0.0745	-8.5	0
IGFBP7	insulin-like growth factor binding protein 7	-3.4	0.0138	-7.2	0
RNASET2	ribonuclease T2	-3.0	0.032	-7.4	0
SAT	spermidine/spermine N1-acetyltransferase	-3.7	0.0073	-7.6	0
BHLHB3	basic helix-loop-helix domain containing, class B, 3	-5.8	0.0002	-2.3	0.04
SELENBP1	selenium binding protein 1	-4.0	0.0033	-4.8	0
C11orf8	chromosome 11 open reading frame 8	-5.0	0.0004	-3.6	0
LSP1	lymphocyte-specific protein 1	-4.9	0.0004	-2.9	0
KRT19	keratin 19	-3.3	0.0	-10.1	0

t* Average t-statistic

q** Average q-value

### Correlation between Rat and Human Data

**Figure 2:**
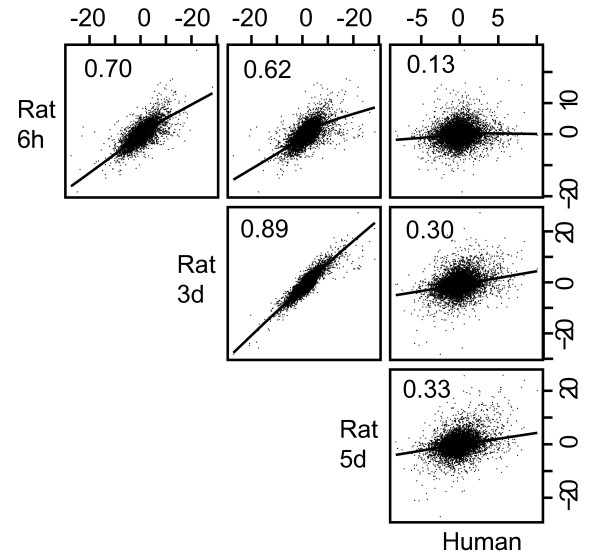
**Correlation of t-statistics.** T-statistics were calculated for estrogen treated samples vs. control (see Methods) for the 9093 clusters of probe sets for both human and rat tissues. Scatter plots of the t-statistic for each gene are shown. Numbers represent correlation coefficients for each respective pair of samples.

In order to compare human and rat vaginal responses to 17β-estradiol we first constructed a per-gene score of differential expression ("*t*^gene^"), by computing the average t-statistic over the probe sets querying a given gene for each of the four datasets (human data, and 3 rat time points). When these *t*^gene ^values for orthologous genes were compared via scatterplot, we observed an overall positive correlation between the four datasets (Figure 2). Pearson correlation coefficients between human and rat were 0.13, 0.30 and 0.33 for the 6 hour, 3 day and 5 day rat data, respectively. This level of positive correlation was statistically significant (see Methods), suggesting that the two species have a similar response to estradiol. Furthermore, it clearly showed that the 5-day time point is more correlated to the human data than is either the 3-day or 6 hour time points. Since the human study involved treatment for 3 months, it likely represents an end stage while the lower correlation to the 6 hr and 3-day time point suggests that these likely represent transition stages. Therefore the 5-day time point was used for further comparisons with the human data.

### Sigpathway analysis

**Figure 3:**
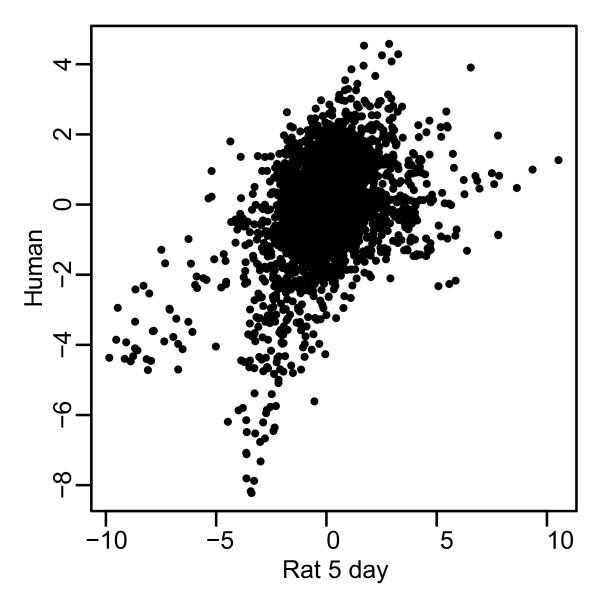
**Gene set correlation.** A scatterplot of the average t-statistic of estradiol treated vaginal tissue compared to untreated vaginal tissue for each of the human gene sets compared to the average t-statistic for each of the rat gene sets. Each point represents a distinct gene set. A positive correlation is seen suggesting many of the gene sets are coordinately regulated. Gene sets in the upper right and lower left corners represent gene sets that are significantly regulated in both rat and human.

**Figure 4:**
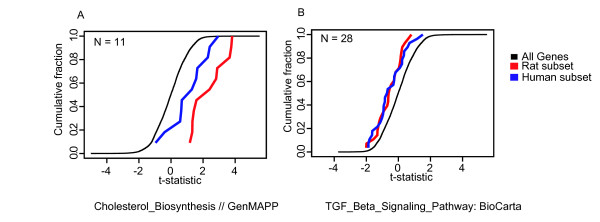
**Cumulative distribution plots.** Plotted is the normalized t-statistics for differential expression on the x-axis plotted against the fraction of the gene set that has that x-value or lower on the y-axis. Gene sets that are shifted away from the plot of the entire data set are considered to be significant. (A) Genes involved in TGFβ signaling are down regulated in both species. (B) Genes involved in cholesterol biosynthesis are up regulated in both species.

We applied a modified version of the Sigpathway algorithm [20] to identify gene sets or pathways significantly regulated by estradiol in either species. This method detects the differential expression of pre-defined gene sets. A gene set is simply defined as a collection of genes sharing a common biological attribute, such as Gene Ontology annotation, signaling pathway membership, or co-expression in another transcriptional profiling dataset. We tested the gene sets from the MSigDB database (version 1.0, Whitehead Institute)[26] for differential expression after estradiol treatment. The analysis was run on the *t*^gene ^values for the 9039 genes with orthologs across species and resulted in 45 gene sets that were significantly regulated in both the human biopsy samples and the rat 5 day treatment samples (Figure 3 and Table 4). Upregulated gene sets include pathways such as cholesterol biosynthesis, steroid metabolism, lysosome function, and epidermis and ectoderm development biosynthesis. Down regulated genes are representative of the TGF-beta signaling pathway, complement activation and matrix metalloproteinases. Examples of two estradiol-sensitive pathways, shown as cumulative distribution plots, are shown in Figure 4. The full list of co-regulated pathways can be found in Table 4.

**Table 4:** Enriched pathways of estrogen regulated genes in human and rat

Gene Set	Rat T1*	Human T1*
Cholesterol_Biosynthesis // Genmapp	6.55	3.91
Cellular Lipid Metabolism // GO:0044255	2.21	3.67
Mitotic Cell Cycle // GO:0000278	2.8	3.14
Cysteine Protease Inhibitor Activity // GO:0004869	2.94	3.02
Small Protein Conjugating Enzyme Activity // GO:0008639	2.34	2.85
Epidermis Development // GO:0008544	5.44	2.65
Steroid Metabolism // GO:0008202	3.23	2.65
Ubiquitin Conjugating Enzyme Activity // GO:0004840	2.3	2.55
Tgf_Beta_Signaling_Pathway: Biocarta	-3.09	-2.54
Complement_Activation_Classical	-2.29	-2.57
Chr3Q13: Cytogenetic Band	-2.36	-2.6
Complement_Activation_Classical // GenMAPP	-2.43	-2.63
TGF_Beta_Signaling_Pathway // GenMAPP	-2.93	-2.64
Matrix_Metalloproteinases // GenMAPP	-3.06	-2.65
Complement Activation, Classical Pathway // GO:0006958	-2.28	-2.67
Cell Differentiation // GO:0030154	-2.39	-2.71
GLUT_UP: Peng_at_al_2002	-3.26	-2.73
chr1q24: Cytogenetic band	-2.87	-2.89
Matrix_Metalloproteinases: BioCarta	-2.52	-2.95
RAP_UP: Peng_at_al_2002	-3.46	-2.99
Locomotory Behavior // GO:0007626	-2.28	-3.14
Transmembrane Receptor Protein Tyrosine Kinase Activity // GO:0004714	-2.66	-3.16
EMT_UP: Jechlinger_et_al_2003	-3.18	-3.21
Specific Rna Polymerase Ii Transcription Factor Activity // GO:0003704	-3.01	-3.24
RAR_UP: Manually Curated	-3.08	-3.25
Response To Wounding // GO:0009611	-2.48	-3.28
Cell Motility // GO:0006928	-2.73	-3.29
Locomotion // GO:0040011	-2.7	-3.33
cell_adhesion	-2.32	-3.34
classic complement pathway	-2.77	-3.34
Vascular Endothelial Growth Factor Receptor Activity // GO:0005021	-2.56	-3.38
Transmembrane Receptor Protein Kinase Activity // GO:0019199	-3.09	-3.48
Immune Complement Pathway	-3.25	-3.66
STTTCRNTTT: IRF:Interferon regulatory factor	-3.55	-3.7
CR_IMMUNE_FUNCTION: PNAS_2009	-3.29	-3.72
Response To Virus // GO:0009615	-2.65	-3.85
Cell Adhesion // GO:0007155	-5.01	-4.05
Humoral Defense Mechanism // GO:0016064	-2.75	-4.37
Humoral Immune Response // GO:0006959	-2.93	-4.42
Antigen Processing // GO:0030333	-3.72	-4.49
morf1_34033_s_at_LILRB1: na	-3.27	-4.66
Antigen Presentation // GO:0019882	-3.98	-5.87
Response To External Biotic Stimulus // GO:0043207	-2.73	-5.95
Response To Pest, Pathogen Or Parasite // GO:0009613	-2.87	-6.21
Immune Response // GO:0006955	-3.61	-6.49

T1* Score of differential expression (NT_k statistic as described in Tian et al[20].)

### Experimental validation of betacellulin

**Figure 5:**
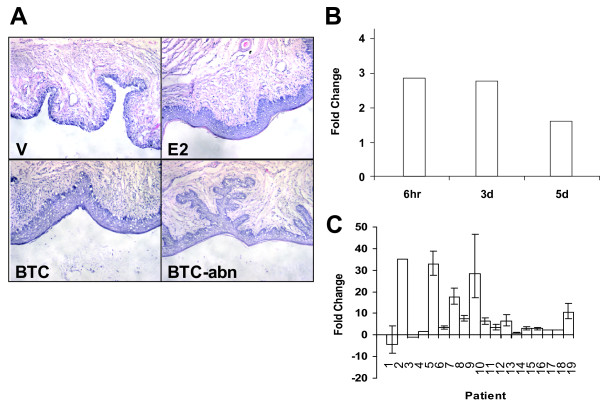
**Expression of BTC increases proliferation of vaginal epithelia.** (A) Hematoxylin and eosin staining of vaginal epithelium from OVX rats treated with vehicle (V), 17β-estradiol (E2), betacellulin with normal histology (BTC) and betacellulin with abnormal histology (BTC-abn). (B) Rat BTC expression levels from microarray analysis at the 6 hour, 3 day and 5 day timepoints. (C) Human BTC expression levels from qRT-PCR analysis versus patient number.

We hypothesized that genes co-regulated in both species are more likely to be important for estrogen signaling and the mitogenic effects seen with estrogen treatment. Further experiments were conducted in the OVX rat model for specific genes of interest, first to validate the microarray results, and second, to determine if the gene product can provide an estrogen-like response. Betacellulin (BTC) is one such gene that was chosen for further validation (Figure 5A). BTC is a widely expressed member of the EGF superfamily and is a known mitogen for a variety of cell types (see [27] for review). It has long been known that EGF can activate ERα and that these signaling pathways converge (see [28] for review), thus this protein was a good candidate for further evaluation.

BTC expression levels increase significantly after estrogen treatment in both rat (Figure 5B) and human. Because expression levels of BTC on the human array were relatively low, qRT-PCR was used to confirm regulation in the human (Figure 5C). BTC is upregulated in 14 of the 17 patients examined. Therefore, it appears that BTC has similar regulation in rat and human. Administration of BTC protein intravaginally in OVX rats results in a thickening and proliferation of the vaginal epithelium as detected by histology (Figure 5A, panel BTC). BTC administration was also noted in some cases to induce abnormal vaginal epithelial proliferation (Figure 5, panel BTC-abn) consistent with its mitogenic role.

## Discussion

The current animal model of VA involves the use of OVX rats. Since the ovaries are the major source of estrogens, removal of the ovaries mimics an estrogen-deprived state. It is well documented that as women enter menopause, the levels of circulating estrogens decrease, resulting in a number of physiological changes, including VA. VA can be reversed through estrogen hormone replacement, suggesting that loss of estrogen is the major factor leading to VA. OVX animal systems mimic many human postmenopausal systems, in both phenotype and histology, and have been well-characterized models for studying these changes. We have taken a systematic molecular approach to determine if the animal model for VA is similar to the human disorder at the transcriptional level.

We have used this experiment to identify genes and pathways that are estrogen regulated in both rat and human vaginal tissue. It is important to note that there are differences in the experimental conditions. First, we evaluated the animal model after 6 hours, 3 or 5 days of treatment. At the 5-day time point, the vaginal epithelium has fully responded to estrogen treatment by histology. We have not, however, compared the transcriptional profiles of normal vaginal tissue across the estrus cycle to vaginal tissue from an OVX animal treated with an estrogen. It is possible that there are differences between the estrogen treated, OVX-animals vs. ovary-intact animals. Second, there have been no comparable studies done in postmenopausal women to determine the time course of histological changes following estrogen therapy. As a result, we chose to look at a time point (3 months) in which we would be sure to see a significant effect. Due to differences in collection times, it is quite possible that we are looking at slightly different molecular endpoints between the human and the OVX rat. Third, it is also worth noting that the tissues being profiled are different. In the animal model we examined the entire vaginal vault, while the human samples contained primarily the vaginal epithelia. Estrogen response likely depends on the cell type composition.

Furthermore, because the rat transcriptome is less well annotated than human, inaccurate or incomplete mappings between the two chipsets may result. Our methods involved grouping together human probe sets mapping to the same gene, calculating the average of their t-statistics, and comparing this value to the average t-statistic for the orthologous rat gene. This procedure was chosen after observing that this metric yielded greater agreement between human and rat in a kidney-vs-liver comparison (see below). However, averaging the results for probe sets querying the same gene could obscure splice-variant-specific responses to estrogen, or dilute desirable signal with poorly performing probe sets. Given the above caveats, it is still encouraging that we find a significant positive correlation in regulation between the two species, suggesting similar underlying molecular mechanisms for estrogen response. This study is also consistent with other validations of this animal model in the literature. OVX animals have long been used to model the effects of estrogens on the reproductive tract and skeleton, and have been validated through a number of different criteria, including histology and response to clinically active agents.

While there is a significant positive correlation of the 17β-estradiol response in rat and human, it should be noted that the correlation is not perfect. There are many possible technical reasons for the differences as described above. To put this in perspective, we performed an identical analysis, where instead of comparing pre- vs post- estrogen treatment, we compared kidney vs liver samples in each species. A comparison between two very distinct tissues represented an idealized dataset, where the results should be the same for human and rat, and also a large degree of differential expression is expected, due to tissue-specific genes. In this regard, we considered the degree of agreement between human and rat for this tissue comparison to be the upper limit of what can be expected. The t-statistic correlation between species was 0.48 (analogous to Figure 2). While 0.48 is higher than the 0.33 we see in our experiments, this is not very surprising since a treatment response within a single tissue is a subtle effect, relative to a comparison between two different tissues. Furthermore, McCarroll et al. [29] studied the transcriptional response to aging in flies vs. worms. They found a t-statistic correlation 0.15 which they note to be highly significant. We expect to have a higher correlation coefficient than 15% since rat and human are evolutionarily closer than flies and worms. Therefore, we feel the ~30% correlation coefficient is in the expected value range (between 15% and 48%).

We were initially surprised that the majority of genes regulated by 17β-estradiol were suppressed rather than stimulated. Our assumption was that 17β-estradiol would exert its pro-proliferatory activities primarily by upregulating genes. However, our data are in line with those obtained from microarray studies done in MCF-7 breast cancer cells, where estrogens also have a pro-proliferatory effect: 70% of the regulated genes were suppressed [30].

Estrogen is the gold standard therapy for treatment of VA and the identification of estrogen regulated genes in both human and rat likely yields genes responsible for the estrogenic effect. We have chosen to validate a gene that is estrogen-responsive in both rat and human vaginal samples. Betacellulin is a member of the epidermal growth factor family of peptide ligands and is a potent mitogen for a wide variety of cell types affecting cell proliferation, differentiation, survival and migration. Estrogens are known to stimulate expression of other members of this family, such as amphiregulin [30]. It has functional redundancy to other members of the EGF family [27]. The increased epithelial thickening seen in the OVX vagina is consistent with its mitogenic role and suggests that BTC may have a normal role in estrogen-dependent epithelial maintenance. Additionally, BTC is able to stimulate mammary gland differentiation as well as promote the survival of breast cancer cell lines [31,32]. However, the uncontrolled proliferation seen in the OVX treated vagina suggests that 17β-estradiol may be regulating other factors to control BTC's mitogenic effect.

## Conclusion

Here we have identified estrogen-regulated genes in the vagina of humans and a commonly used rat animal model. In addition to demonstrating that the vagina is a highly estrogen-responsive organ at the molecular level, we show that the transcriptional response to estrogen is highly correlated between the two species. Furthermore, we hypothesized that genes with estrogen-sensitivity in both species are more likely to be responsible for the changes observed in estrogen treated OVX rats. We have shown that BTC, a gene regulated by estrogen in both human and rat, does in fact have the ability to recapitulate the estrogen induced epithelial proliferation. It is likely that many other genes also possess the ability to induce epithelial proliferation and current investigations are underway to identify them.

## Abbreviations

OVX: ovariectomized; E2: 17β-estradiol; BTC: betacellulin; VA: Vaginal Atrophy; VMI: Vaginal maturation index; GO: genome ontology; KEGG: Kyoto Encyclopedia of Genes and Genomes; migdb: mitochondrial interest group database; EGF: epidermal growth factor; ERα: estrogen receptor alpha; GCOS: Affymetrix gene chip operating system 1.0.

## Competing interests

All authors work for Wyeth Research. Patents related to this research have been applied for.

## Authors' contributions

SAJ participated in the design, analyzed the expression data and drafted the manuscript. SEC analyzed the expression data and performed the statistical analysis. JSC carried out the betacellulin studies and helped to draft the manuscript. MMC designed the human clinical trial. EW performed the microarray assays. KS performed the qPCR reactions. AJD participated in the design of the study. ELB participated in the design of the study. BJP participated in the betacellulin studies. XZ participated in the betacellulin studies. RCW participated in the design of the study. HAH conceived the study, participated in the design and helped to draft the manuscript. All authors read and approved the final manuscript.

## Pre-publication history

The pre-publication history for this paper can be accessed here:


